# Reply: Concerns regarding the study “Comparing the effects of a commercial and a prototype vitrification medium on meiotic spindle morphology and survival rate of mouse oocytes”

**DOI:** 10.5935/1518-0557.20230016

**Published:** 2023

**Authors:** Iara Gonçalves Roberto Viana, Alessandra Aparecida Vireque, Paula Andrea Navarro

**Affiliations:** 1 Division of Human Reproduction, Department of Gynecology and Obstetrics, Ribeirao Preto Medical School, University of São Paulo, Ribeirão Preto, SP, Brazil; 2 Semear Fertility Clinic, Ribeirão Preto, SP, Brazil; 3 Invitra Assisted Reproductive Technologies LTD., Ribeirão Preto, SP, Brazil; 4 National Institute of Hormones and Women’s Health CNPq, Brazil

We agree with the author of the letter regarding our paper that researchers should follow the science rules, and, in this way, it is important to mention that all authors have decades of experience on the theme cryopreservation and are doing good science (Da Luz *et al*., 2022; Berteli *et al*., 2022a; Vireque *et al*., 2016; Xie *et al*., 2021; de Lima *et al*., 2018), as working in innovative research in human assisted reproductive technologies (Invitra 2017; 2018; 2022; Viana *et al*., 2018), generating invention patents (Vireque *et al*., 2013; 2018; 2019), and contributing therefore with scientific and technological advances in our country. Always focusing on the best scientific practices.

The author of the letter regarding our paper states that the prototype Tvitri-4 is a “homemade” medium, but this assertion does not correspond to reality. Tvitri-4 was developed by the biotech Invitra, a spin-out of University of São Paulo, founded in 2013 and that initiated its main laboratory activities in 2016 supported by two FAPESP research grants for Innovative Research in Small Business (PIPE) (Invitra, 2017), a RHAE Researcher in Company grant from Brazilian Programme on Human Resources for Strategic Areas/RHAE/CNPq (Invitra, 2018), and a Productivity Scholarship in Technological Development and Innovative Extension CNPq (Invitra, 2022). Besides, all steps of the Tvitri-4 medium development were performed in a controlled environment in a laboratory with state-of-the-art equipment and standard operational procedures.

According to the references cited in our paper, there is not a defined standard for the minimum or maximum number of oocytes to be vitrified per straw. In addition, there is no such indication in the study published by Almodin *et al*. (2010), nor in the manufacturer’s protocol of Vitri-Inga, which describes all the instructions on how to vitrify and handle the vitrification media and straws. It is important to note that according to Almodin *et al*. (2010), the oocyte survival rate was 84.9%, and, in our study, it was 81.5%, so very similar results. Wang *et al*. (2013), used a vitrification protocol similar to that of the present study and had very good warming results, reporting 94-95.1% of survival rates. The first author of our paper is an experienced senior embryologist with 3000 vitrification procedures performed over several years of work in the area (4130 oocyte collection procedures; 5852 intracytoplasmic sperm injection; 3605 embryo transfer and 3157 embryo biopsy). Besides, her experience was always updated in many hands-on vitrification courses at Brazilian congresses, offered by different IVF companies including the Ingamed group (Pre-congress course, XIII Brazilian Congress of Assisted 2009; Oocyte vitrification course, XXVII Brazilian Congress of Human Reproduction, 2016).

In the article from Almodin *et al*. (2010), the exact volume of DV-I solution used in the warming procedure was not described. In addition, the results of our study do not differ markedly from those found by Almodin *et al*. (2010). Some warming techniques were already tested differing in relation to the volume of medium used in the first thawing solution (TS), in which the samples remain 1 min at 37ºC. Wang *et al*. (2013) used a volume of 1ml and their results were 94-95.1%. De Munck *et al*. (2013) compared TS volumes much smaller than 1 ml (25µl vs. 150µl) and reported survival rates of 65.9% and 89.6%, respectively. It should be noted that using a TS volume of 150µL, remarcably lower than 0.5-1mL, the volume range highlighted in the current letter, an oocyte survival rate higher than that found by Almodin *et al*. (2010) was obtained.

Regarding the Tvitri-4 formulation, it is surprising that the author of the letter on our paper is not familiar with the process of intelectual property protection. The prototype Tvitri-4, developed by Invitra, is protected by an industrial secret, a modality of intellectual property (IP) rights on confidential information which may be sold or licensed. This was the recommendation of the innovation agents and the IP legal office that advises us. Moreover, Invitra in partnership with the University of São Paulo is co-owner of an invention patent filed in 2019 intitled “Vitrification and/or warming composition, process for improving cell cryopreservation, and use of the vitrifying and/or warming composition, 2019, Brazil. BR1020190136979”(Vireque *et al*., 2019). In this same year, the authors of the article “Comparing the effects of a commercial and a prototype vitrification medium on meiotic spindle morphology and survival rate of mouse oocytes” received the Professor Nilson Donadio award, conferred by the Sociedade Paulista de Medicina Reprodutiva in recognition of innovation in oocyte vitrification media. In 2021, our research group was honored again with the Soledad Sepulveda Award from the Red Latinoamericana de Reproducción Asistida for the comprehensive analysis of the membrane lipid profile of vitrified and/or IVF oocytes and embryos (Berteli *et al*., 2022b).

It also should be noted that in ongoing studies by our research group focused on supplementing vitrification media with fatty acids and L-carnitine, the Tvitri-4 medium has also shown excellent performance compared to the Irvine Scientific vitrification medium as a control, regarding the oocyte membrane lipids profile and IVF outcomes (Berteli *et al*., 2022a).
